# Selection of Aptamers for Mature White Adipocytes by Cell SELEX Using Flow Cytometry

**DOI:** 10.1371/journal.pone.0097747

**Published:** 2014-05-20

**Authors:** Eun Young Kim, Ji Won Kim, Won Kon Kim, Baek Soo Han, Sung Goo Park, Bong Hyun Chung, Sang Chul Lee, Kwang-Hee Bae

**Affiliations:** 1 Research Center for Integrated Cellulomics, KRIBB, Daejeon, Republic of Korea; 2 Medical Proteomics Research Center, KRIBB, Daejeon, Republic of Korea; 3 BioNanotechnology Research Center, Bioconvergence Research Institute, KRIBB, Daejeon, Republic of Korea; 4 Department of Functional Genomics, University of Science and Technology (UST) of Korea, Daejeon, Republic of Korea; University of São Paulo, Brazil

## Abstract

**Background:**

Adipose tissue, mainly composed of adipocytes, plays an important role in metabolism by regulating energy homeostasis. Obesity is primarily caused by an abundance of adipose tissue. Therefore, specific targeting of adipose tissue is critical during the treatment of obesity, and plays a major role in overcoming it. However, the knowledge of cell-surface markers specific to adipocytes is limited.

**Methods and Results:**

We applied the CELL SELEX (Systematic Evolution of Ligands by EXponential enrichment) method using flow cytometry to isolate molecular probes for specific recognition of adipocytes. The aptamer library, a mixture of FITC-tagged single-stranded random DNAs, is used as a source for acquiring molecular probes. With the increasing number of selection cycles, there was a steady increase in the fluorescence intensity toward mature adipocytes. Through 12 rounds of SELEX, enriched aptamers showing specific recognition toward mature 3T3-L1 adipocyte cells were isolated. Among these, two aptamers (MA-33 and 91) were able to selectively bind to mature adipocytes with an equilibrium dissociation constant (*K*d) in the nanomolar range. These aptamers did not bind to preadipocytes or other cell lines (such as HeLa, HEK-293, or C2C12 cells). Additionally, it was confirmed that MA-33 and 91 can distinguish between mature primary white and primary brown adipocytes.

**Conclusions:**

These selected aptamers have the potential to be applied as markers for detecting mature white adipocytes and monitoring adipogenesis, and could emerge as an important tool in the treatment of obesity.

## Introduction

In our present-day society, many people suffer from various metabolic disorders [1]. Obesity is associated with many metabolic disorders and is certainly one of society’s most controversial contemporary issues. It leads to type II diabetes, dyslipidemia and hypertension, and constitutes an increased risk for the development of cardiovascular diseases [2]; [3]. From the results of multiple studies, scientists have suggested various therapeutic methods related to obesity, such as bariatric surgery, anti-obesity medications (orlistat, rimonabant and sibutramine) and stem cell therapy [4]–[6]. Nevertheless, the development of more effective methods is still required for treating obesity, as all of the existing methods have potential side effects [7]; [8]. A specific molecular probe against mature adipocyte cells would be valuable for mitigating potential side effects.

Aptamers have been widely applied in the diagnosis and treatment of various diseases. These are oligonucleic acid molecules that have specific three-dimensional structures for recognizing their target molecules, which can be proteins, chemicals or heterogeneous cells, among others [9]–[11]. Aptamers were isolated from a library pool (oligonucleic acids; initial pool size of 10^14∼15^) by the Systemic Evolution of Ligands by EXponential enrichment (SELEX) method. The SELEX technique was developed by Dr. Larry Gold, who selected RNA ligands against the T4 DNA polymerase using repeated rounds of *in vitro* selection [12]. In 1990, the SELEX technique was upgraded to forms of a Spiegelmer, cell SELEX capillary electrophoresis SELEX (CE-SELEX), Counter-SELEX, and Toggle SELEX [13]–[17]. Macugen, the first aptamer-based drug approved by the U.S Food and Drug Administration (FDA), is offered by OSI Pharmaceuticals and used as a therapeutic agent for age-related macular degeneration (AMD) [18]. In addition, NeoVentures Biotechnology Inc. has successfully commercialized the first aptamer-based diagnostic kit for the detection of mycotoxins in grains. At present, several aptamers are considered to be therapeutic or diagnostic agents and are undergoing clinical trials [19].

Given this background, the SELEX technique can be used for the diagnosis of white adipocyte density/replication. Adipose tissue, mainly composed of adipocytes, is an important metabolic organ, which serves as a modulator of energy homeostasis [20]. Obesity is induced when the energy balance is broken in the body. In mammals, adipose tissue is typically classified into white adipose tissue (WAT) and brown adipose tissue (BAT) according to its functions and morphological appearance [21]. WAT is used as a store of extra energy, and the cells contain a single large lipid droplet. BAT, a specialized form of adipose tissue, can generate heat for energy consumption as a thermogenic organ. Brown adipose cells contain multiple smaller lipid droplets. Based on these characteristics, the development of obesity is closely related to the differentiation of white adipocytes [22]. The techniques employed in the diagnosis of obesity include physical examination, blood test, body mass index (BMI), and skin fold test using X-ray technique or a body average density measurement.

In this study, we attempted to isolate specific aptamers that specifically recognize mature adipocyte cells using 3T3-L1 cells, which is considered as a typical cell line of white preadipocytes. Subsequently, we selected two aptamers, which specifically bind to mature white adipocytes, by the cell SELEX method using a FACS. Even the isolated aptamers were able to distinguish primary white adipocytes from primary brown adipocytes. These aptamers can be applied as valuable tools for a variety of anti-obesity approaches.

## Materials and Methods

### Ethics Statement

All procedures used in animal experiments were performed according to a protocol approved by the Animal Care and Use Committee of the Korea Research Institute of Bioscience and Biotechnology (Permit Number: KRIBB-ACE-13047). All surgery was performed under ether anesthesia, and all efforts were made to minimize suffering.

### Aptamer Library and Primers

A single-stranded DNA (ssDNA) library was labeled with a fluorescein isothiocyanate (FITC) and synthesized by Integrated DNA Technologies, Inc. (Coralville, IA, USA). The library contained 40 random nucleotides (nt) flanked by two 19-nt primer hybridization sites (5′-FITC-CGCGGAAGCGTGCTGGGCC-N_40_-CATAACCCAGAGGTCGAT-3′). For the amplification of the selected aptamer pool, a FITC-labeled forward primer (5′-FITC-GGGGAATTCGCGGAAGCGTGCTGGGCC-3′) and a reverse primer (5′-GGGGGGATCCATCGACCTCTGGGTTATG-3′) were used in the PCR process. Another forward primer (5′- GGGGAATTCGCGGAAGCGTGCTGGGCC-3′) was utilized for the cloning of the selected DNA pool. Additional nucleotide sequences (containing restriction enzyme sites) were added in forward and reverse primers for efficient subcloning.

### Cell SELEX Using FACS

To start the procedures, we prepared preadipocytes (0 day; negative cells) and mature adipocytes (12-day cultured cells in a differentiation medium; target cells) of 3T3-L1 cells. In the first SELEX, the FITC-labeled ssDNA library (10 nmol; initial pool size 10^16^) dissolved in 20 µL DW was denatured at 95°C for 5 min and then cooled on ice for 10 min to form a secondary structure. During this step, salmon sperm DNA (0.1 mg/mL; Sigma, St Louis, MO, USA) dissolved in 300 µL of binding buffer (4.5 g/L glucose, 5 mM MgCl_2_ and 10% FBS in Dulbecco’s PBS [Sigma]) was incubated with 1×10^6^ target cells to inhibit non-specific binding. Next, the cells were incubated with the ssDNA library (10 nmol) and bovine serum albumin (1 mg/mL; Thermo Inc., Rockford, IL, USA) at 37°C for 30 min. After centrifugation, the supernatant was removed and the cells were washed five times with 1 mL of binding buffer. The ssDNA library-bound cells were then enriched using a FACSAria cell sorter (BD Bioscience, San Jose, CA, USA) and the bound ssDNAs were eluted from the sorted cells by heating at 95°C for 5 min. The eluted DNAs were purified by phenol-chloroform (Sigma) extraction, a Sephadex G-25 column (Sigma) and ethanol precipitation. The purified ssDNAs were amplified by PCR with FITC-labeled primers. For the next round of selection, the single-stranded DNA population was obtained via strand separation of PCR products (heat treatment at 95°C for 10 min). After five rounds of selection, the binding time and treated concentration of the ssDNA pool were decreased respectively to 15 min and 10 pmol, while the other conditions were maintained until the final SELEX round (12 rounds). Negative selection was performed two times during the third and eighth rounds of SELEX. To ensure the identification of extremely specific aptamers for mature 3T3-L1 cells, we performed three additional SELEX rounds using a nine-round SELEX pool. The ssDNA pool of twelfth round was cloned into DH5α using a TA cloning kit and was sequenced (Enzynomics, Daejeon, Korea). A total of 12 rounds of SELEX were performed, and two aptamers, MA-33 and MA-91, were obtained.

### Aptamer Binding Assay by FACS and Confocal Microscopic Imaging

To monitor the enrichment of aptamer pools during SELEX, a 10 pmol ssDNA pool of each round was incubated with 1×10^6^ mature adipocyte cells in 200 µL of binding buffer at 37°C for 7 min. Selected aptamers were incubated with various cell lines under the same condition. The cells were washed five times with 0.4 mL of binding buffer. The pellets were suspended in 20 µL of binding buffer, and 10 µL of cells were dropped on a glass slide. The FITC signal of the aptamers was detected with a LSM 510 META confocal microscope (Zeiss, Thornwood, NY, USA). The other half of the cells was resuspended in 0.2 mL of binding buffer and the fluorescence was determined with a FACS Calibur flow cytometer (BD Bioscience).

### Competition Binding Assay

Both FITC-labeled and unlabeled aptamers for MA-33 and MA-91 were used for competition binding assay. The 10-fold excess unlabeled aptamer (4.5 µM) was incubated with mature 3T3-L1 cells at 37°C for 7 min. After preincubation of the unlabeled aptamer, the other FITC-labeled aptamer (450 nM) was added. After incubation at 37°C for 7 min, the cells were washed five times with washing buffer, and the fluorescence was determined with a FACS Calibur flow cytometer (BD Bioscience).

### Structure Prediction and *K*d Determination

Secondary structures of the aptamers were predicted using the *mfold* program (The RNA Institute) [23]. We chose aptamers with the most thermodynamically stable predicted structure after the sequencing of each aptamer. Individual aptamers were incubated with negative or positive cells. As the treated aptamers increased in number, the mean fluorescence intensity of the aptamer-coated cells was detected using FACS. Next, the equation *Y* = *B*_max_X/(*K*d+X) was used to calculate the equilibrium dissociation constant (*K*d) of aptamer-cell interaction via SigmaPlot (Jandel, San Rafael, CA, USA) (X; radioligand concentration, Y; specific binding, and B_max_; maximum number of binding sites).

### Cell Culture and Adipogenic Differentiation

The 3T3-L1 is a preadipocyte cell line derived from mouse embryonic fibroblasts, and was purchased from ATCC. The preadipocyte cells were cultured in a growth medium (high-glucose DMEM containing a 1% antibiotic-antimycotic solution and 10% bovine calf serum; Gibco-Invitrogen, Carlsbad, CA, USA) in a 5% CO_2_ incubator at 37°C. C2C12, HeLa, and HEK-293 cells were grown in a growth medium with 10% FBS. Adipogenic differentiation was induced as described in our previous reports [24]–[29]. Confluent 3T3-L1 cells were treated with high-glucose DMEM, 10% FBS, and MDI (a differentiation cocktail of 0.5 mM 3-isobutyl-1-methylxanthine, 1 µM dexamethasone, and 10 µg/mL insulin [Sigma]) for 2 days. The cells were then cultured in high-glucose DMEM, 10% FBS, and 10 µg/mL insulin until day 12. The medium was changed every 2 days.

### Isolation, Culture, and Differentiation of Primary White and Brown Preadipocytes

White preadipocytes were isolated from subcutaneous areas (inguinal and epididymal fat) and visceral adipose tissue (mesenteric fat) of 8-wk-old male ICR mice (Jackson Laboratory, Bar Harbor, ME, USA). Additionally, brown preadipocytes were extracted from interscapular brown fat pads of mice. The dissected tissues were digested for 45 min at 37°C in an isolation buffer at pH 7.4 (123 mM NaCl, 5 mM KCl, 1.3 mM CaCl_2_, 5 mM glucose, 100 mM HEPES, 4% filtered BSA, and 1 mg/mL collagenase type II [Worthington, Lakewood, NJ, USA]). The undigested tissues were then removed using a 100-µm cell strainer, after which the remaining cells were centrifuged at 1,300 rpm for 3 min to pellet the white or brown preadipocytes. The white preadipocyte cells were resuspended in DMEM/F12 [1∶1; Gibco-Invitrogen] containing a 1% antibiotic-antimycotic solution and 10% BCS. Confluent cells were exposed to MDI containing 1 µM rosiglitazone in DMEM/F12. After 2 days, the cells were maintained in DMEM/F12 with 10 µg/mL insulin and 1 µM rosiglitazone (Sigma) for 2 days in each case for 20 days [30]. Isolated brown preadipocytes were induced to differentiate into mature brown adipocytes, as described previously [31]; [32]. For differentiation, confluent brown preadipocytes were placed in high-glucose DMEM, 20% FBS, and a differentiation cocktail (0.5 mM 3-isobutyl-1-methylxanthine, 125 µM indomethacin, 0.5 µM dexamethasone, 20 nM insulin, and 1 nM 3, 3′,5-triiodo-L-thyronine [T_3_] [Sigma]) for 2 days. Next, the medium was changed to a maintenance medium consisting of DMEM, 20% FBS, 1 nM T_3_ (Sigma), and 20 nM insulin, which was refreshed every other day.

### Oil-Red-O Staining

After washing twice with PBS, the cultured cells were fixed with 10% formalin for 30 min at room temperature. The cells were then washed with distilled water and stained with a 0.3% filtered Oil-Red-O solution in 60% isopropanol (Sigma) for 30 min at room temperature. The stained cells were washed five times with distilled water and then dried. To extract the combined Oil-Red-O dye, isopropanol was added to the stained cells. The extracted samples were detected at 510 nm using a GeneQuant 1300 spectrophotometer (GE HealthCare, Uppsala, Sweden) [26]–[29]. Micrographs were obtained from triplicate samples.

### Trypsin Treatment of Mature Adipocytes

Mature adipocytes were incubated with trypsin-EDTA (Gibco-Invitrogen) for 5 min. After washing two times with binding buffer, 1×10^6^ cells were treated with 400 nM aptamers (library, MA-33 or MA-91) as described in the binding affinity test section.

## Results

### Enrichment of Aptamers for Mature Adipocytes by CELL SELEX using Flow Cytometry

**Figure 1:**
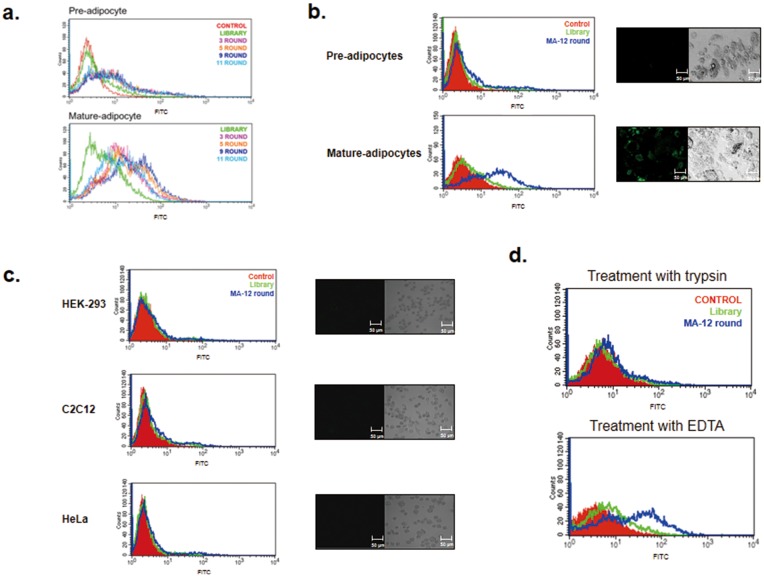
Binding affinity test of each round pool with mature adipocytes as a target cell. **A.** After a total of 11 rounds of SELEX processing, the binding affinities of four representative round pools (3, 5, 9 and 11 rounds) were analyzed for preadipocytes (negative cells) and mature adipocytes (target cells) using flow cytometry. **B.** The MA-12-round pool, a more enriched pool than the MA-9-round pool towards mature adipocytes, was enriched through additional three SELEX rounds using the MA-9-round pool (see Fig. S2). The binding affinity of MA-12-round pool was analyzed for preadipocytes (negative cells) and mature adipocytes (target cells) using flow cytometry and confocal imaging. **C.** The binding specificity of the MA-12-round aptamers for various cell lines was verified by using three types of cell lines (HEK-293, C2C12, and HeLa cells). Each cell line was incubated with a library or with the MA-12-round pool. Next, the cells were monitored by FACS (left) and confocal imaging (right). **D.** To examine the binding of MA-12-round aptamers towards trypsin-treated target cells, differentiated 3T3-L1 cells were treated with trypsin or 2% EDTA. Then, the cells were incubated with MA-12-round pool and were analyzed by FACS.

The mature adipocytes and preadipocytes from 3T3-L1, a typical cell line for investigating adipocytes *in vitro*, were used for positive and negative selection, respectively (Fig. S1). After the induction of adipogenic differentiation, the cell morphological patterns and expression levels of the adipogenic markers (aP2, adiponectin, PPARγ, resistin and C/EBPα) were examined by FACS, western blotting, and real-time PCR analysis [29]; [33]. Then, to obtain aptamer candidates for mature adipocyte cells, we applied the CELL SELEX method using flow cytometry (we termed this method FACS-SELEX) [5]; [34], which is a typical cell-based SELEX method. The random 40-mer single-stranded DNA (ssDNA) library was used to select specific aptamers toward mature adipocytes. The amplified ssDNA pool after each round of selection was used for the next round of SELEX. Negative selection was attempted two times against preadipocyte cells during the third and eighth rounds. After 11 rounds of selection, four representative round pools (3, 5, 9 and 11 rounds) were evaluated for their binding affinity towards mature adipocyte cells. In general, increasing the number of SELEX rounds led to a gradual increase in the fluorescence intensity towards mature adipocytes, and specific aptamers with better binding affinity to mature adipocyte cells were enriched in each round pool. However, the peak shift displayed a tendency to move back to the library peak during round 11 (Fig. 1A lower panel). On the other hand, the peak shift was not detected with preadipocyte cells (Fig. 1A upper panel). To ensure extremely specific aptamers for mature 3T3-L1 cells, we performed an additional three SELEX rounds using a nine-round SELEX pool. It is known that the SSC-H (side scatter) value of flow cytometry is well correlated with the degree of adipogenic differentiation [33]; [35]. Thus, differentiated adipocytes were incubated in a nine-round pool and the cells were analyzed and sorted by FACS (Fig. S2). The aptamer pool for the next round was obtained from only fully mature adipocytes showing high SSC-H values (see P3 region of Fig. S3-C).

The specificity of the aptamer pool after 12 rounds of SELEX was examined using various cell lines, in this case HEK-293, C2C12, and HeLa cells by FACS as well as a confocal image analysis (Figs. 1B and 1C). As shown in the left panel of Figs. 1B and 1C, the peak shift of the 12-round aptamer pool was only detected in mature 3T3-L1 adipocyte cells compared to that of the library. A similar result was also observed in the confocal image analysis (see right panel of Figs. 1B and 1C), implying that the aptamer pool after 12 rounds of SELEX is highly specific toward mature adipocyte cells.

To examine if the targets of the 12-round aptamer pool were membrane proteins on the target cells, we broke up the membrane proteins by a trypsin treatment before adding the 12-round aptamer pool [36]. After binding the 12-round aptamer pool to trypsin-treated cells, the peak shift was not detected (Fig. 1D). In contrast, the affinity of the 12-round pool with the target cells was retained when it was treated with 2% EDTA on mature adipocyte cells to conserve the membrane proteins. These results clearly suggested that the majority of the binding partners of the 12-round aptamer pool consisted of membrane proteins on mature adipocyte cells.

### Isolation of Aptamers Specific for Mature Adipocytes from an Enriched 12-round Aptamer Pool

Based on data from the 12-round aptamer pool, we concluded that the 12-round aptamer pool holds a number of potential aptamer candidates with high affinity toward mature adipocytes. Therefore, 91 aptamers were cloned and sequenced from the final-round aptamer pool (Table S1). However, the conserved sequence motif cannot be obtained from sequence alignment analysis. This may be due to the number of targets on the surface of the mature adipocytes. As an initial step, the secondary structure of 91 aptamers was predicted using the *mfold* program (http://mfold.rit.albany.edu). Subsequently, their binding affinity for both pre- and mature adipocytes was analyzed by using FACS. Finally, three aptamers with high affinity for mature adipocytes were isolated and the equilibrium dissociation constants (*K*d) were calculated (Table 1). These aptamers, named MA-33, MA-64, and MA-91, showed affinity towards mature adipocytes in the nanomolar range.

**Table 1:** List of sequences and *K*d values for three selected aptamers.

Aptamers	Sequences	*K*_d_ (nM)
**MA-33**	GTTACCGCGGTGAAGGGTGGATGTGTCTGGACGCTATATC	142.9±3.8
**MA-64**	AAGATGACAAATTGCTGGAGTGTCGTGGCGAGGATGTCGG	213.5±3.2
**MA-91**	CACCGGCAGGCCAAATAACAGGCATCACACACACTGCAGG	33.1±2.9

### Verification of the Specificity of Two Aptamers for Mature Adipocytes

**Figure 2:**
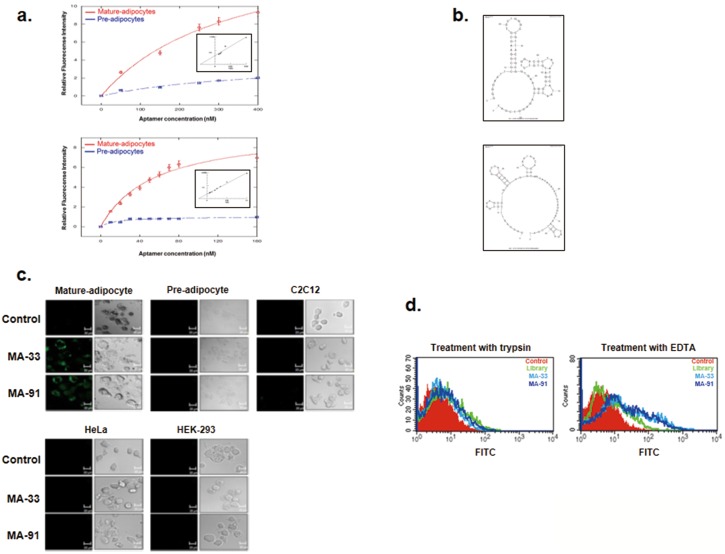
Characterization and binding affinity assays of MA-33 and MA-91. **A.** Along with an increase in the aptamer concentration, the binding degree of the two aptamers towards preadipocytes and mature adipocytes was determined by FACS. **B.** The secondary structures of MA-33 and MA-91 were predicted using the *mfold* program. **C.** Each cell line was incubated with MA-33 and MA-91, and the fluorescence of the cells was then detected with a confocal microscope. Mature adipocytes, preadipocytes, C2C12, HeLa, and HEK-293 cells were used to test whether MA-33 and MA-91 bind specifically to mature adipocytes. In each figure, the left panels illustrate the fluorescence images and right panels illustrate the phase-contrast images. **D.** To confirm whether the binding partners of MA-33 and MA-91 are proteins, mature adipocytes were incubated with trypsin (left) or 2% EDTA (right). The cells were mixed with MA-33 and MA-91. The level of fluorescence was then detected using FACS.

We chose two aptamers with high affinity towards mature adipocytes, and binding assays of these aptamers were performed by using flow cytometry (Fig. S3). The MA-33 and MA-91 aptamers showed very tight binding towards mature adipocytes with *K*d values of 142.9±3.8 nM and 33.1±2.9 nM, respectively (Table 1 and Fig. 2A). On the other hand, these aptamers do not bind to undifferentiated 3T3-L1 preadipocyte cells (Fig. 2A). These results clearly demonstrate that the two selected aptamers efficiently discriminate between preadipocytes and mature adipocytes and specifically bind only with mature adipocyte cells. Fig. 2B shows the secondary structure of MA-33 and MA-91 aptamers predicted using a secondary structure prediction program.

The specificity of these two aptamers was further confirmed in various cell lines, such as HEK-293, C2C12, and HeLa cells, using a confocal microscopic analysis (Fig. 2C). As shown in Fig. 2C, the MA-33 and MA-91 were exclusively bound to mature adipocytes. Next, we tested whether the binding partners of the selected aptamers were membrane protein(s) of the target cells by FACS (Fig. 2D). The results indicated that the peaks of MA-33 and Ma-91 were only moved to the right when mixed with 2% EDTA-treated mature adipocyte cells compared to control cells and trypsin-treated mature adipocyte cells. These results clearly suggested that the target(s) of the two selected aptamers were proteins expressed on the cell surfaces of mature adipocytes.

### Monitoring of MA-33 and MA-91 Binding during Adipogenic Differentiation

**Figure 3:**
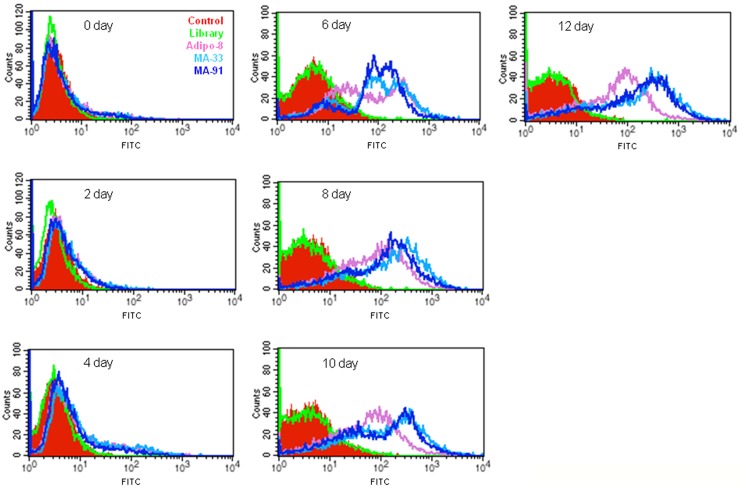
The measurement of MA-33 and MA-91 binding towards 3T3-L1 cells during adipogenic differentiation. The 3T3-L1 cells were harvested at 0, 2, 4, 6, 8, 10s and 12 days after differentiation. The cells were then incubated with MA-33, MA-91s or Adipo-8 aptamers and the binding was examined by flow cytometry.

Next, the time-course monitoring of the aptamer binding process during the adipogenic differentiation of 3T3-L1 cells was monitored. MA-33 or MA-91 was incubated with cells harvested at 0, 2, 4, 6, 8, 10 or 12 days during adipogenic differentiation. Aptamer binding was then assessed using flow cytometry. As shown in Fig. 3, MA-33 and MA-91 did not bind with the cells until 4 days after adipogenic differentiation. However, MA-33 and MA-91 abruptly bound with cells on the 6th day, after which the binding affinity was maintained, and increased, until the late stages of adipogenic differentiation (Fig. 3). The binding patterns of MA-33 and MA-91 were generally similar to each other, but they differed significantly from that of Adipo-8, a previously reported aptamer specific for mature adipocytes. These results indicated that the binding target(s) of MA-33 and MA-91 might differ from that of Adipo-8. Additionally, we carried out binding assay of FITC-labeled MA-91 in the presence of excess unlabeled MA-33 (also *vice versa*). Even though ten-fold excess of unlabeled MA-33 was pretreated with mature adipocytes, it could not influence the binding of FITC-labeled MA-91 (Fig. S5). Therefore, MA-33 and MA-91 were assumed to have the different binding target(s) on the surface of mature white adipocytes. Furthermore, these results clearly imply that the binding of MA-33 and MA-91 is not non-specific toward cell surface proteins, but specific to certain target protein(s).

### Selected Aptamers can Distinguish Primary White Adipocytes from Primary Brown Adipocytes

**Figure 4:**
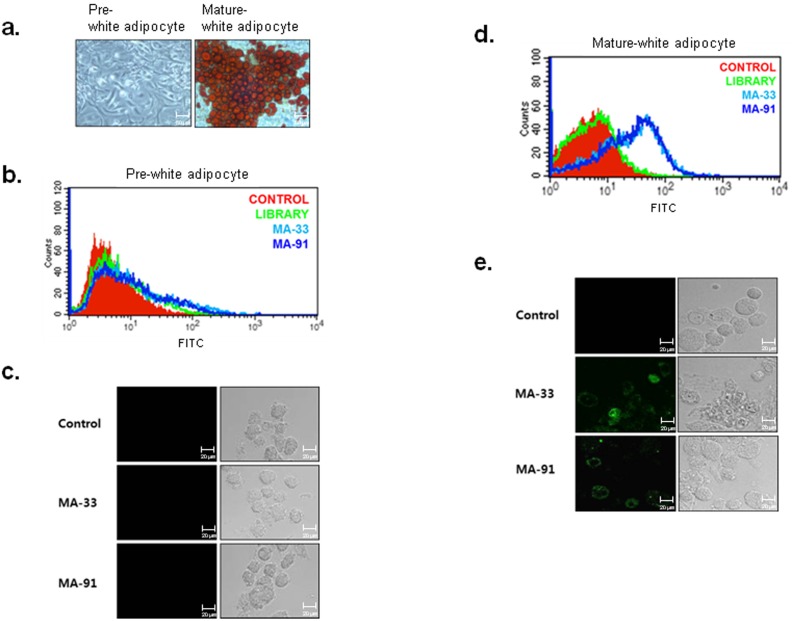
Binding test of selected aptamers towards primary white adipocyte cells. **A.** Primary cultured white preadipocytes were induced to differentiate into mature adipocytes. The lipid droplets of the mature adipocytes were stained with Oil-Red-O. **B.** The binding affinity of MA-33 and MA-91 for pre-white adipocyte was analyzed by FACS. **C.** The binding affinity of MA-33 and MA-91 for pre-white adipocyte was analyzed by a confocal image analysis. **D.** The binding affinity of MA-33 and MA-91 for mature-white adipocyte was analyzed by FACS. **E.** The binding affinity of MA-33 and MA-91 for mature-white adipocyte was analyzed by confocal image analysis.

In this study, we obtained aptamers specific for mature adipocytes using 3T3-L1 preadipocyte cell line. Next, to validate the specific binding of the aptamers toward primary adipocyte cells, we prepared two types of primary adipocyte cells, white and brown adipocytes, from white and brown adipose tissues isolated from ICR mice. White adipocyte precursor cells were separated from both subcutaneous areas (inguinal and epididymal fat) and from the visceral adipose tissue (mesenteric fat) of 8-week-old male ICR mice. The isolated precursor cells were cultured, and the cells were then induced to differentiate into mature white adipocytes. Lipid accumulation was assessed by Oil-Red-O staining (Fig. 4A), which indicated that the isolated precursor cells were well differentiated under our experimental conditions. FITC-labeled MA-33 and MA-91 aptamers were incubated with undifferentiated or differentiated cells, and the fluorescence intensity was detected using FACS and confocal image analysis. The results clearly suggested that MA-33 and MA-91 aptamers bind only to primary mature white adipocyte cells, whereas they do not recognize primary white precursor cells (Figs. 4B to E).

**Figure 5:**
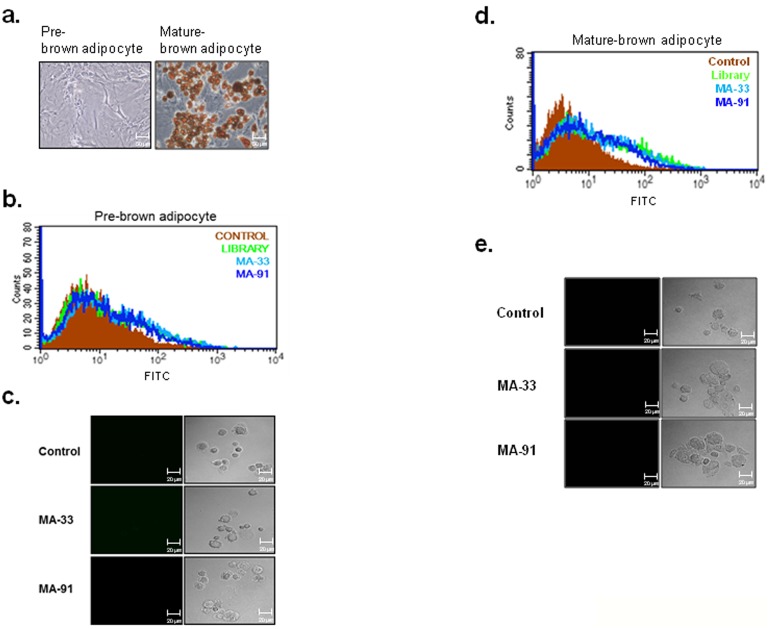
Binding test of selected aptamers toward primary brown adipocytes. **A.** Primary brown preadipocytes were induced to differentiate into mature brown adipocytes. The differentiation process was monitored by staining with Oil-Red-O. **B.** The brown preadipocytes were incubated with MA-33 or MA-91, and the binding affinity was analyzed using FACS. **C.** The brown preadipocytes were incubated with MA-33 or MA-91, and the binding affinity was analyzed by confocal imaging. **D.** The mature brown adipocytes were incubated with MA-33 or MA-91, and the binding affinity was analyzed using FACS. **E.** The mature brown adipocytes were incubated with MA-33 or MA-91, and the binding affinity was analyzed by confocal imaging.

Next, to test the possibility that the selected aptamers can distinguish white mature adipocytes from brown mature adipocytes, MA-33 and MA-91 aptamers were incubated with primary brown adipocyte cells. Brown precursor cells were isolated from the interscapular brown fat pad of mice one day after birth, and were induced to differentiate into mature brown adipocytes by culturing with a brown differentiation medium. The efficient differentiation of brown adipocytes was confirmed by Oil-Red-O staining (Fig. 5A). Unexpectedly, the selected aptamers did not bind to differentiated brown adipocytes, unlike mature white adipocytes (Figs. 5B to E). Based on these results, we concluded that the two selected aptamers could distinguish between white adipocytes and brown adipocytes. In particular, they can specifically detect mature white adipocyte cells. Additionally, the specificity of these aptamers is retained not only in cell lines but also in primary cells.

## Discussion and Conclusion

Many studies have suggested the potential use of aptamers in therapeutic approaches, diagnostic methods, and basic science applications. This has been facilitated by the SELEX technique, repeated rounds of *in vitro* selection, and advancements in using RNA and DNA aptamers since the 1990s [12]; [37]. Since the discovery of aptamers, the SELEX process was modified by various methods and the duration of a selection experiment was reduced from six weeks to three days [38]. In fact, several aptamers are already used as drug delivery system or as diagnostic tools.

Our research goal was to isolate specific aptamers for mature adipocytes. In this study, FITC-labeled ssDNA aptamers were used as a library for the FACS-Cell SELEX process to obtain aptamers specific for mature adipocytes. The 3T3-L1 line, the well-characterized white adipocyte model cell line for studying obesity, was chosen as the target cell line. The transcriptional activation and repression of adipocyte genes were clearly revealed during the progression of 3T3-L1 preadipocyte differentiation [39]; [40]. To obtain aptamers having high specificity to mature adipocytes, negative selection was performed at least two times during a 12-round selection process using 3T3-L1 preadipocytes. The aptamer population with high fluorescence intensity toward mature adipocytes was increased to nine rounds, but this pattern was not shown when assessing the preadipocytes (Fig. 1A). After adipogenic induction, the cells were composed of a heterogeneous population. The granularity of the cells (SSC) gradually increased during differentiation and was well correlated with the degree of differentiation [33]; [35]. Therefore, we performed additional SELEX rounds to obtain more specific aptamers towards mature adipocytes using mature adipocytes having high SSC values (Fig. S3). From a total of 12 rounds of SELEX, high specificity of the aptamer pool of MA-12 round was obtained and confirmed in various cell lines by FACS and a confocal image analysis (Figs. 1B and 1C). One of the most important aspects in this study is the reduction in the numbers of SELEX cycles using FACS sorting [34]. On average, more than 20 cycles of SELEX rounds are required to enrich an aptamer population with high specificity [41]; [42]. For example, Tang et al. [43] have selected a specific aptamer to recognize Ramos cells, a Burkitt’s lymphoma cell line by 23 SELEX round. After 22 rounds of selection, AptTOV1 was selected from ovarian cancer cells as a target cell [44]. However, we enriched and isolated specific aptamers with only 12 SELEX rounds. Adipo-8, a specific aptamer for differentiated 3T3-L1 cells as determined by Liu et al.,[45] was selected via 19 SELEX rounds using Cell SELEX despite the fact that the target cell is identical. A total of 91 candidates were selected after 12 rounds of SELEX, and the binding affinity for preadipocytes and mature adipocytes was investigated. Finally, MA-33 and MA-91 were selected as specific aptamers for mature adipocytes (Fig. S4). The two selected aptamers can distinguish the differentiated 3T3-L1 cells from various cells (Fig. 2C). MA-91, which showed the lowest *K*d value among the three selected candidates, was further tested to determine its properties. We compared the binding affinity of MA-91 with that of Adipo-8 in differentiated 3T3-L1 cells (Fig. S4). Although the binding temperature of Adipo-8 was different from our selection condition, the binding affinity of Adipo-8 was retained at our binding temperature. The affinity of MA-91 was slightly stronger than that of Adipo-8. Furthermore, MA-33 and MA-91 could detect mature primary white adipocytes, but not primary brown adipocytes (both pre- and mature form). This indicates that our aptamers can detect proteins expressed only on the membrane of mature white adipocytes. Additionally, we anticipate that the target protein(s) of aptamers can be used as a biomarker(s) for mature white adipocytes and for this reason they are valuable as a target(s) for monitoring and regulating obesity [46]; [47]. Until recently, liposuction is the preferred treatment that removes fat from various parts on the human body [48]. Like any major surgery, this technique also carries various risks such as contour irregularities, fat embolism and bleeding [49]. One of the biggest risk factors is the inability to specifically distinguish fat tissue from various tissues. Many different liposuction skills have been developed for reducing side effects, but they are not perfect solutions. In this study, we suggest that MA-33 and 91 will be a new way to solve this problem. Furthermore, we isolated the aptamers at 37°C, indicating the high potential for *in vivo* applications [50]. Therefore two aptamers of this study are expected to be available against obesity care. Considering the results obtained thus far, MA-33 and MA-91 can both be used as aptabody (a biomarker probe for a target cell to replace an antibody) for mature white adipocytes. They are anticipated to have a wide range of applications in the following areas. (1) Since most membrane proteins are receptor-types, MA-33 and MA-91 may inhibit the receptor signaling related to adipogenic differentiation [51]. For example, insulin signaling regulates glucose homeostasis and the energy balance by lipid storage in adipose tissue [52]; [53]. Signaling occurs via the insulin receptor, and aberrant signaling results in the clinical manifestation of obesity, diabetes, and different cancers. In fact, mice with fat-specific disruption of the insulin receptor gene have low fat mass levels, experience a loss of body weight, and undergo obesity-related glucose intolerance [54]. (2) Our study indicates that specific delivery of anti-obesity drug(s) to mature white adipocytes by targeted vehicles via the internalization characteristics of aptamer(s) is possible [55]. (3) This approach can also be used to obtain homogeneous adipocytes for both basic research and for clinical applications [56]. At present, we are examining whether the selected aptamers can bind to a third class of adipocytes called brite adipocytes (known as beige cells) [57]–[61]. Brite adipocytes are catagorized as brown adipocyte-like cells, which reside in some white adipose tissues. In conclusion, we isolated two aptamers with highly specific binding activity towards mature white adipocytes. These aptamers can be valuable as potential tools for basic and clinical approaches related to anti-obesity treatment.

## Supporting Information

Figure S1 **Schematic presentation of FACS-Cell SELEX used to isolate the aptamer(s) for mature adipocytes.** This method integrates the Cell SELEX technique with FACS sorting system. To monitor the enrichment of aptamer pools during SELEX, FITC-labeled ssDNA library (10^14^∼10^16^) was incubated with target cells (pre-adipocytes or mature-adipocytes). Then, the ssDNA library-bound cells were sorted using FACS. For the next round, purified ssDNA was amplified by PCR with FITC-labeled primers. We repeated this process for enrichment of aptamers. After the final round of SELEX, aptamer candidates were identified by cloning and sequencing.
(TIF)

Figure S2 **Additional SELEX rounds were performed with the quantified mature adipocytes.** The characteristics of the differentiated 3T3-L1 cells were determined using forward scatter (FITC; x-axis) versus side-scatter (y-axis) during FACS analysis. The differentiated 3T3-L1 cells (A) were incubated with a library (B) or the MA-9-round pool (C). The cells were divided into three sections according to the side-scatter values (P1<P2<P3; differentiation degree) on dot plots. The aptamers were isolated and eluted from only the P3 region, and this was then amplified by PCR for the next round. We repeated this process three times to create the MA-12-round pool.
(TIF)

Figure S3 **Binding test of two selected aptamers.** Ninety-one candidates in total were sequenced, and the binding affinity levels for preadipocytes and mature adipocytes were analyzed. Among the aptamers tested, MA-33 (A) and MA-91 (B) were selected based on their specificity and affinity characteristics.
(TIF)

Figure S4 **Comparative experimental studies among MA-33, MA-91, and Adipo-8.** Mature 3T3-L1 cells were incubated with MA-33, MA-91, or Adipo-8. The binding affinity levels were then confirmed using FACS analysis.
(TIF)

Figure S5 **Competitive binding assay between MA-33 and MA-91.** FITC-labeled MA-91 aptamer was incubated with 10-fold excess unlabeled MA-33. Then, the binding of MA-91 toward mature adipocyte cells was measured using FACS analysis.
(TIF)

Table S1 (DOCX)
