# Supplementary figures and images for: Selection of Aptamers for Mature White Adipocytes by Cell SELEX Using Flow Cytometry
Source: PLoS One. 2014 May 20;9(5):e97747. doi: 10.1371/journal.pone.0097747 (PMC4028271; doi:10.1371/journal.pone.0097747)

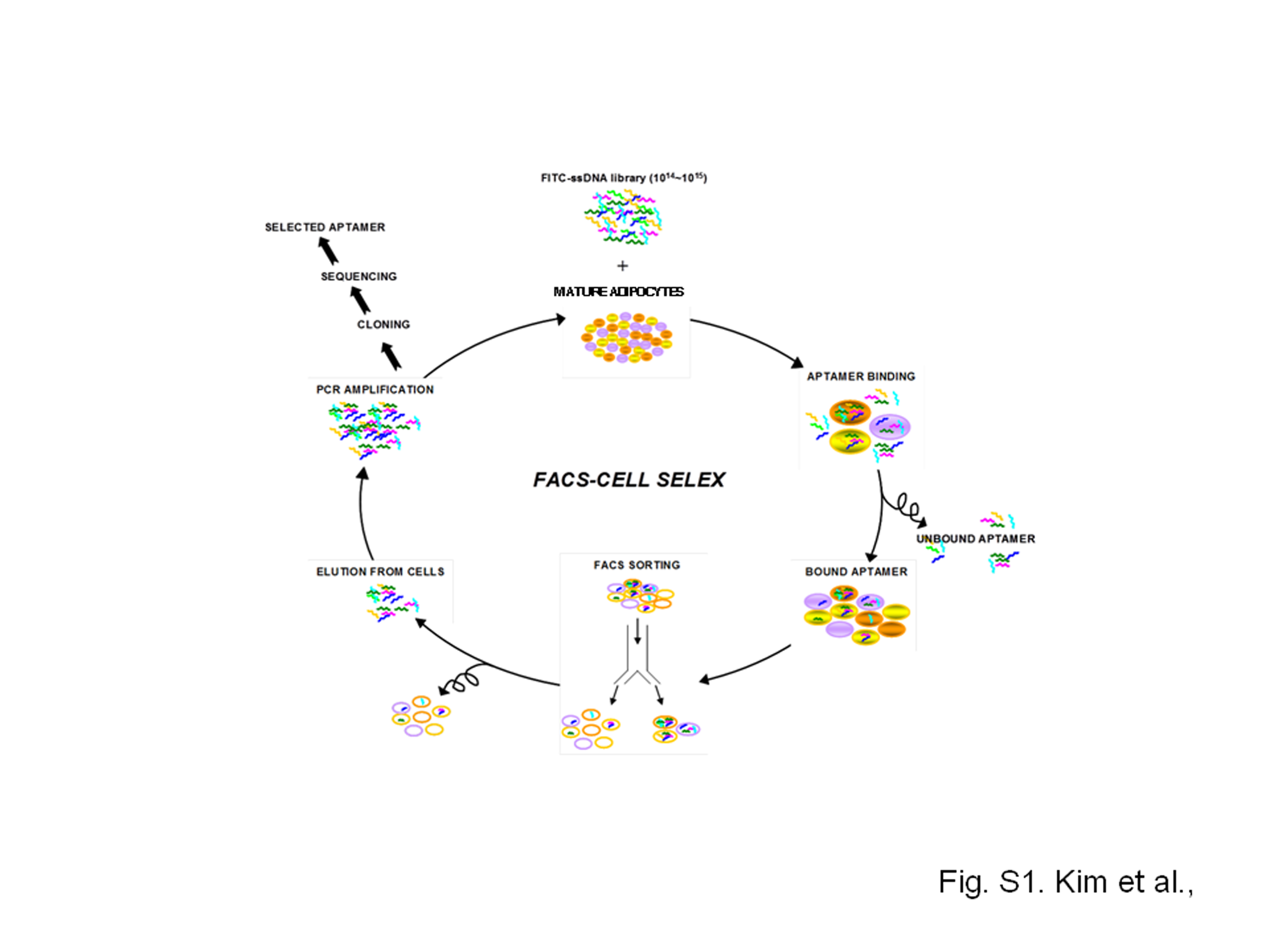

Supplement: Figure S1 — Schematic presentation of FACS-Cell SELEX used to isolate the aptamer(s) for mature adipocytes. This method integrates the Cell SELEX technique with FACS sorting system. To monitor the enrichment of aptamer pools during SELEX, FITC-labeled ssDNA library (1014∼1016) was incubated with target cells (pre-adipocytes or mature-adipocytes). Then, the ssDNA library-bound cells were sorted using FACS. For the next round, purified ssDNA was amplified by PCR with FITC-labeled primers. We repeated this process for enrichment of aptamers. After the final round of SELEX, aptamer candidates were identified by cloning and sequencing. (TIF) [file pone.0097747.s001.tif]

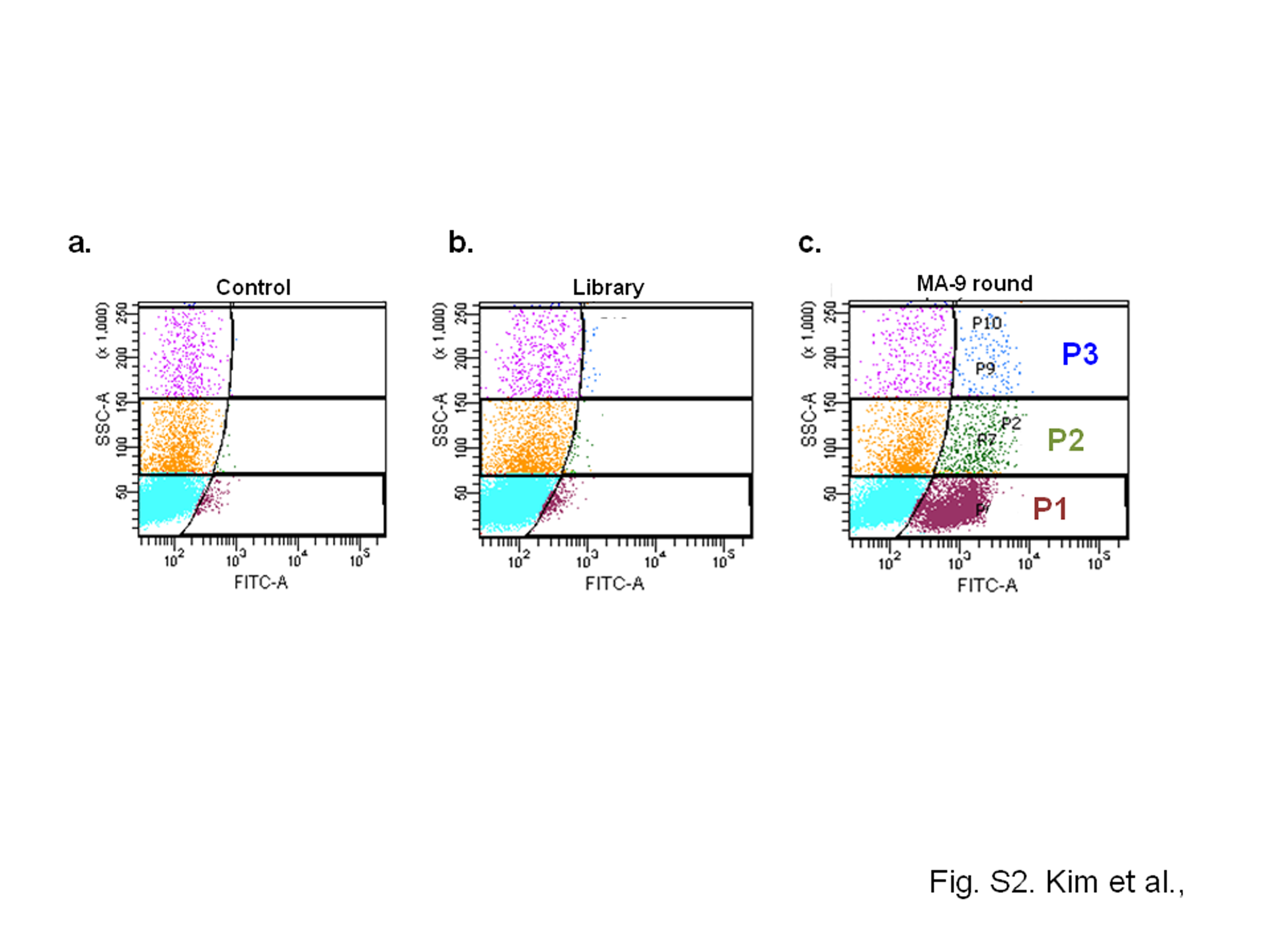

Supplement: Figure S2 — Additional SELEX rounds were performed with the quantified mature adipocytes. The characteristics of the differentiated 3T3-L1 cells were determined using forward scatter (FITC; x-axis) versus side-scatter (y-axis) during FACS analysis. The differentiated 3T3-L1 cells (A) were incubated with a library (B) or the MA-9-round pool (C). The cells were divided into three sections according to the side-scatter values (P1<P2<P3; differentiation degree) on dot plots. The aptamers were isolated and eluted from only the P3 region, and this was then amplified by PCR for the next round. We repeated this process three times to create the MA-12-round pool. (TIF) [file pone.0097747.s002.tif]

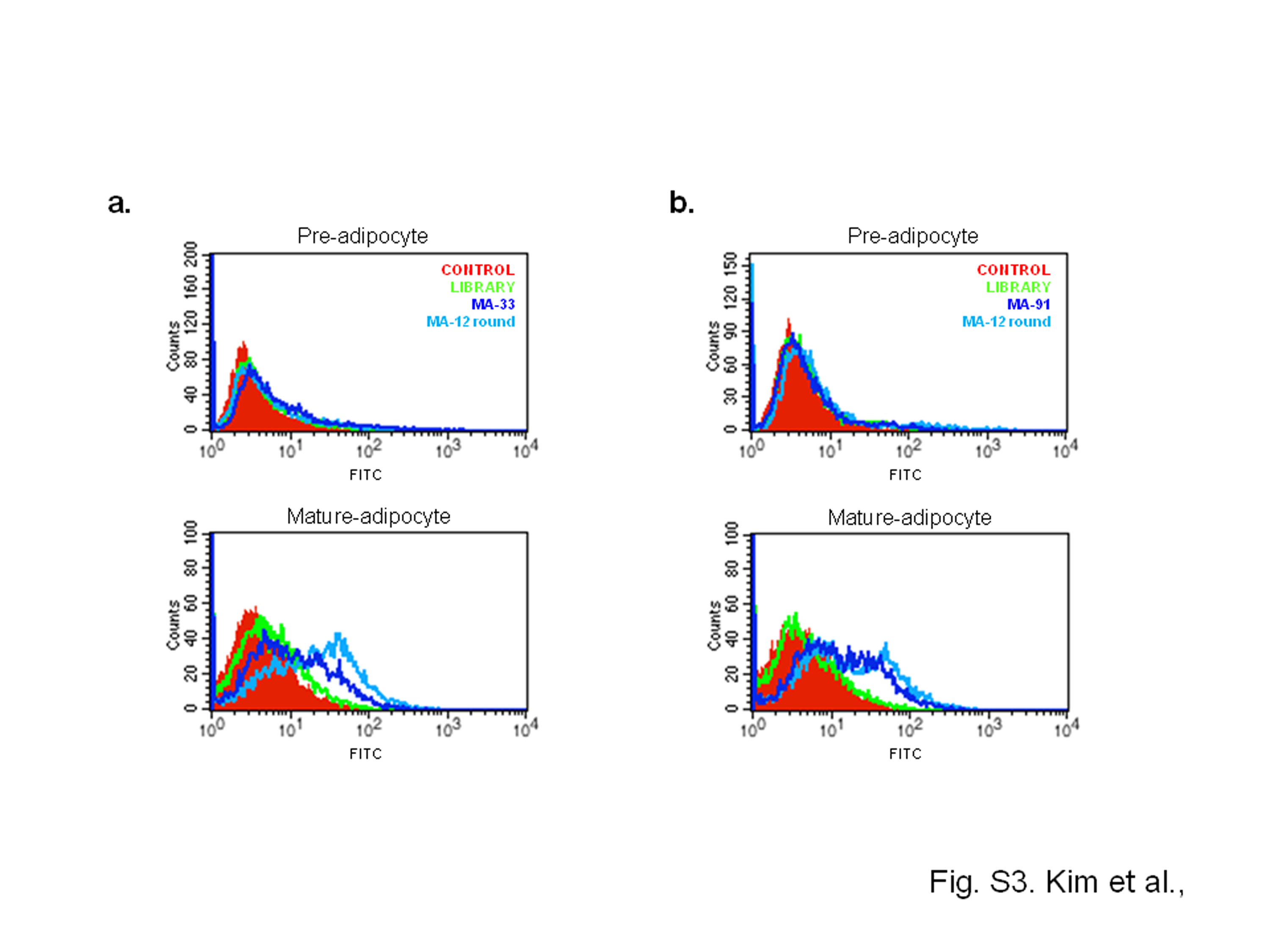

Supplement: Figure S3 — Binding test of two selected aptamers. Ninety-one candidates in total were sequenced, and the binding affinity levels for preadipocytes and mature adipocytes were analyzed. Among the aptamers tested, MA-33 (A) and MA-91 (B) were selected based on their specificity and affinity characteristics. (TIF) [file pone.0097747.s003.tif]

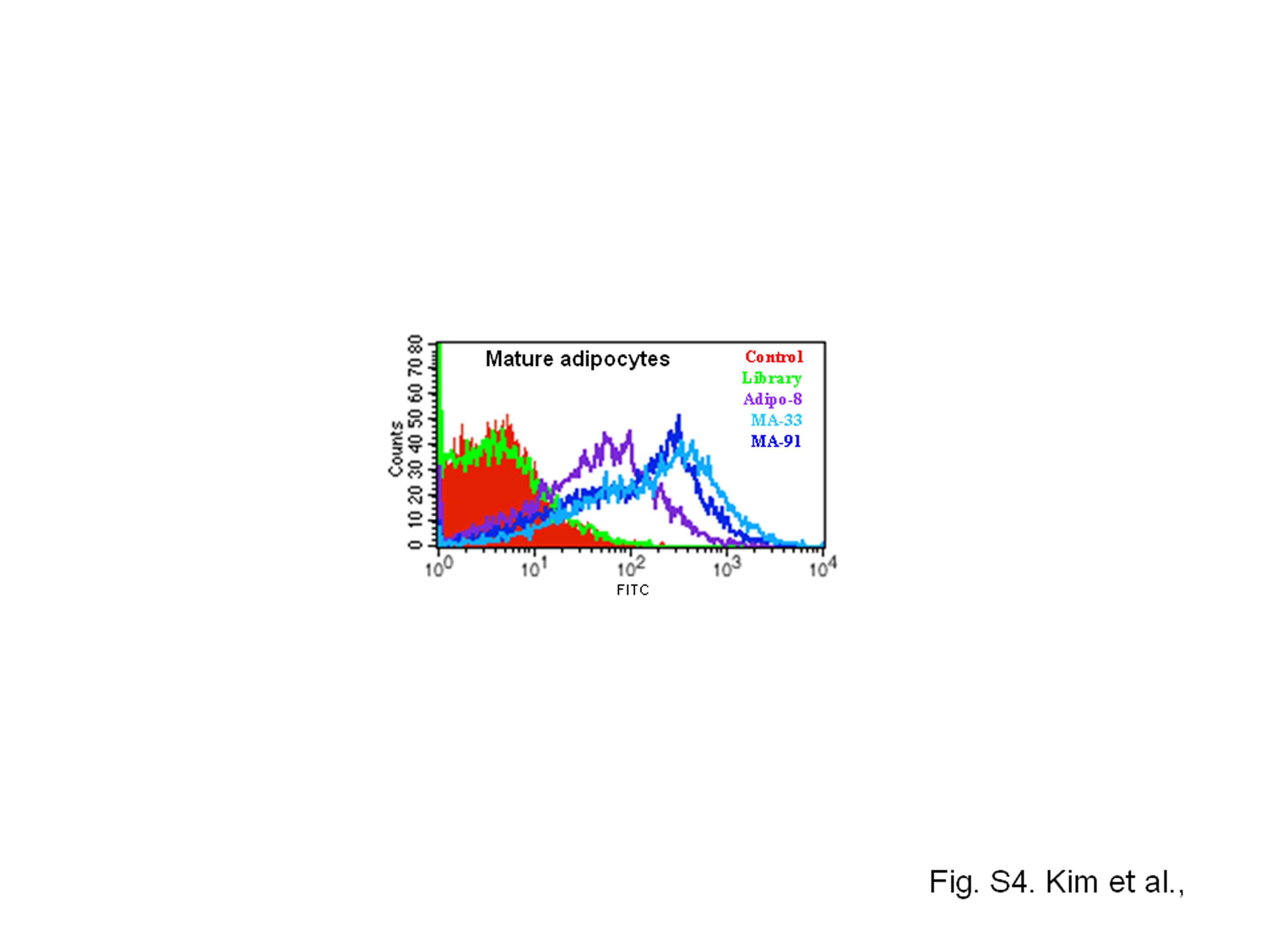

Supplement: Figure S4 — Comparative experimental studies among MA-33, MA-91, and Adipo-8. Mature 3T3-L1 cells were incubated with MA-33, MA-91, or Adipo-8. The binding affinity levels were then confirmed using FACS analysis. (TIF) [file pone.0097747.s004.tif]

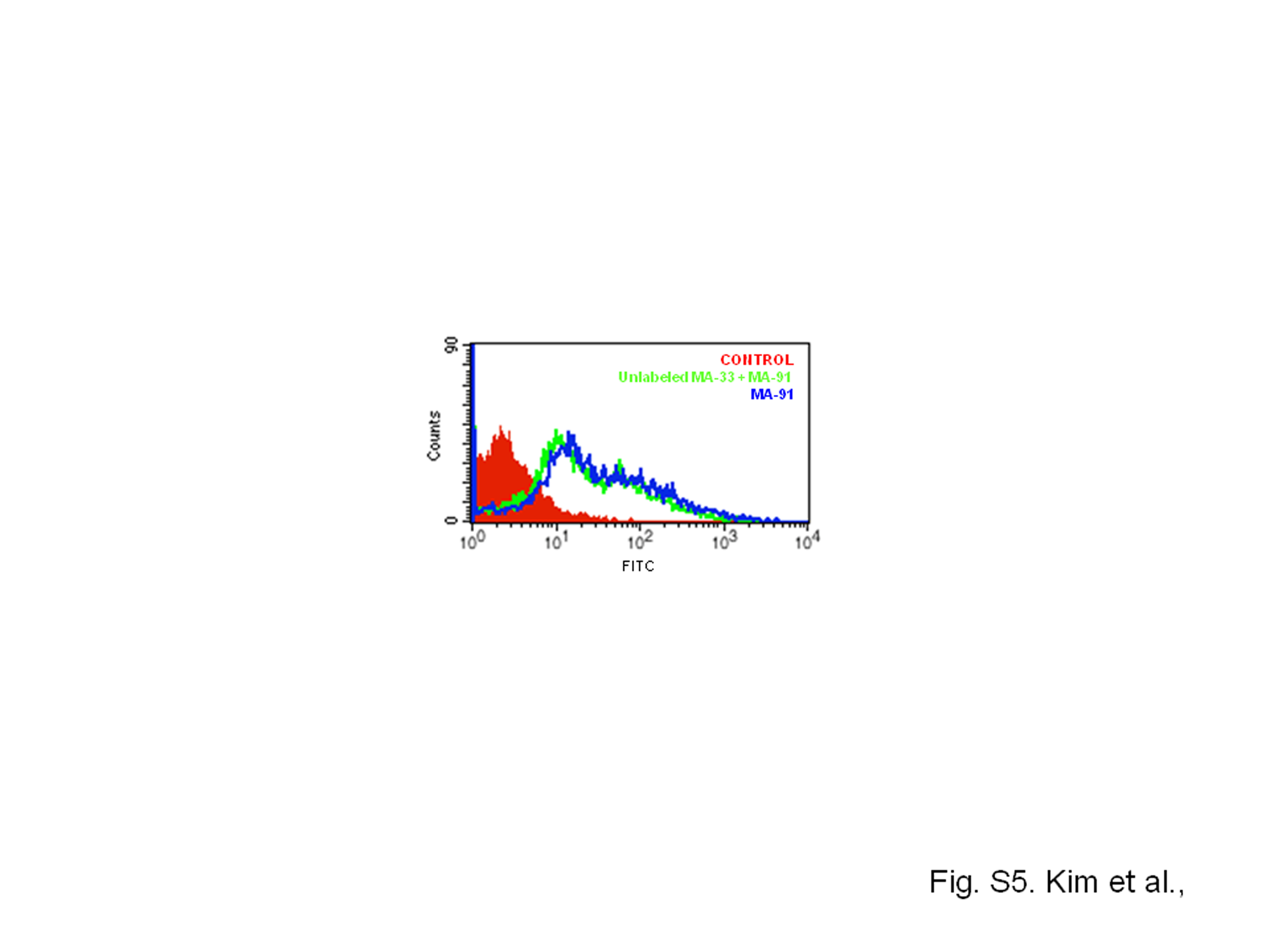

Supplement: Figure S5 — Competitive binding assay between MA-33 and MA-91. FITC-labeled MA-91 aptamer was incubated with 10-fold excess unlabeled MA-33. Then, the binding of MA-91 toward mature adipocyte cells was measured using FACS analysis. (TIF) [file pone.0097747.s005.tif]
