# Supplementary material for: Selection of Aptamers for Mature White Adipocytes by Cell SELEX Using Flow Cytometry
Source: PLoS One. 2014 May 20;9(5):e97747. doi: 10.1371/journal.pone.0097747 (PMC4028271; doi:10.1371/journal.pone.0097747)
Supplement: Table S1 — (DOCX) [file pone.0097747.s006.docx]

**Table S1.** Sequences of selected aptamers to mature adipocytes.

| NAME | Sequence (5’-3’) |
| --- | --- |
| MA-01 | GAAAATGCCACACACTCGTCTAAACTCGCCAATCTCAGCA |
| MA-02 | TTATTTCAGCCGACGGAACTGACGGACAGAGCTCCTTACA |
| MA-03 | AAAGCCTCGATCCTGACCTTAACTCCTATGACGTCGTGTA |
| MA-04 | TTGAGAATGCTGGCGAGAAGGTTGCTGACGCTCGTGTGGC |
| MA-05 | GTGGTGATCCGAGTCCAGGCCCCAGTCCCATTGCATTAGT |
| MA-06 | AACGGTGGACCAGTGTGGTAAAGATTGACTGTGGCGGGAC |
| MA-07 | GTCTTTTTAGTCTGGGCAATGCAGGTGTGTTAGTCCTCTG |
| MA-08 | AACATGAAACAACGGCGGCGTGCACTGCGGACTGATGACT |
| MA-09 | GGTGAGAGAATTATAAGAGGACGGGATTCAGGGCGCTAGT |
| MA-10 | GCGGTTTGGAACTCCGTGGTGTGTGCGACTACCTTTTTGC |
| MA-11 | GTTTCTGGCCATTTGACTTCTTTCAGGAAAACTGGACGGA |
| MA-12 | ACTATTGAACGGCAGGGCGTAGTAGGTTTGGCCGGGAGCA |
| MA-13 | TCAACCAGCACCCCAACCACATCGTGTATCCATTGCCGCT |
| MA-14 | CGAGACCACCTTATCTACTCGAATCCCACACGTATGCACT |
| MA-15 | GGTGTGTCTTCTTTGAGTCTGACTTGGTGCTGTGTGAGGC |
| MA-16 | GTCTCACACCGCGCGCCTAACATCAGTGCCAGCAAAAGCC |
| MA-17 | GCCAAACTGCTGAACGAAATGATTTCCGAGGATTAAGACA |
| MA-18 | GTTTGTGCTGCTATAGGTCTACGGTGACATATCGTGAGGA |
| MA-19 | AACGCGCACGCTCCGATAGAAACCCATCTTACTATAGCAA |
| MA-20 | GCACTGTCACTAATTTTGATTACGCACCTCCTACTGAAAG |
| MA-21 | GAGCCAAGACAATGCACATTTCACACCGGTATATTGTTCG |
| MA-22 | ACAAACATTCCATGCGTACAGAAAGGAACTCAAAGGTATA |
| MA-23 | GTTCCGTCTTTACGTTGAGAAATGTGACTGAGCTCGTACT |
| MA-24 | TCGCGTAGATGGATATGGATGTGGACTTGCAGGGTCGTTG |
| MA-25 | ATGCTGATTCCCGTGGTCCAAAACACTCACTCGCCACTGA |
| MA-26 | GCTGACTGTTTTCTACCGGTTTTAATGTGCTAGAGTGGAG |
| MA-27 | GTCCGAACCCCCACAATAAATTGCTAAAAAGGTGTGTTT |
| MA-28 | AACTGTGACGCTGGCATGGTGGCTCATCGGTCTTCAAATC |
| MA-29 | GCGATAGTACAGAGTTGGTCCCGAGGAGTCAGTCATATTT |
| MA-30 | TCGCCGTTCACTGATTCTTTCTTGTTAATGTTTGCCACAT |
| MA-31 | TGACCCACTATCCGACTTAACGCTATCCTCAAGATGAACA |
| MA-32 | TGTTCCGAGAAATTTTAGCTGAGGCTTTGAACGTACATTA |
| MA-33 | GTTACCGCGGTGAAGGGTGGATGTGTCTGGACGCTATATC |
| MA-34 | CGATAGAGTTGTTGCGATTAGATATGTTGCAGGTTTGCCG |
| MA-35 | CGTGGTTACGTTTTCCTCGTCGTGCCTAAGTCCGAATCCT |
| MA-36 | ACCTTTGTCAACGAAGTTAGTGGCGGGATGGTAGGTGAGG |
| MA-37 | AACGACAGAACAAGGTGGATTTGCGCCTAGGACATTTTA |
| MA-38 | TTGGTAGTTCTGTGTTAGCGGGTACTTATCGGCGTCGGGA |
| MA-39 | TAACGAGTTTACCGCTCTGTAGGGCTTAACTGTCAGGCAA |
| MA-40 | CGAAACGTCAGAATCATCAATTCACAGTCCTCATCAGCCA |
| MA-41 | GATTTACTAAGACGACTGCTTGAGAAACACACAACTTTGA |
| MA-42 | ACTTGTCCTTAGTTGTCCCGCAATTACACCTTATACCTCA |
| MA-43 | GACTCCTTTCCCAATCTAGTTACAGCAGACGACAAATTAC |
| MA-44 | TGAATGTGTTTGCGTGAGAAGTGATTTGGCTAGACGTGCG |
| MA-45 | TACTGCCGTTTATAAGACGCCCTATGCCTTTTTTCATTAA |
| MA-46 | TGTGCCTAGTGCCTGGCGTCGGTACTTGTAAAGCTGAACT |
| MA-47 | TGAGTACGAGTTGGCCAGTGGCTTTCCTGTGTGAGGGATA |
| MA-48 | CCGATCATACATGATAAAGATAGCTGGTGTGAGTGCGATG |
| MA-49 | TTAAACCCAATCTGTTGTCGGAGTATACGGATGGGAGGTG |
| MA-50 | CGTATTTACACTCTACATCTGACCATACAATCTGCATCTT |
| MA-51 | GGATAAACGATATGTCACATAGTTAAGGGTCGGACGGGGG |
| MA-52 | GTCTCGTTCATCATTCTGCAGGGCTCTTGGATAACACTTA |
| MA-53 | ATAAATTCAATTAAATCTCATACTCCGTGACTGCAAGTTT |
| MA-54 | AAGAGGTGCTTTTGCGTTGGATTTTCTCTCTGAGCGGCAG |
| MA-55 | GAGGCGTTTTAGTTTAATGATGTGTGGGGCATTGTGCGTG |
| MA-56 | TCCGTTGTGCAGTCAATGTAAGGGTGTCAAGTAATTTGTT |
| MA-57 | ACTTCAAGTCGGTTAAAGCGTTACCATGCCGGCTAGTTGT |
| MA-58 | AGCCTATTAAGCCTACTCCACTAAGTTGTGATACTGAACA |
| MA-59 | GTAGATTAGTGCGACAGGGGGGCTACGAAGATGAAACTGG |
| MA-60 | CACCCAACCAAACGTTATCATCGGTGCGGTAGTATTGCCT |
| MA-61 | GCCGGCCGATTCTGTAGCCCCACTTCTACCTTTTAAGCCG |
| MA-62 | AAGAACACCCCCTATATCTATTTCAACGCCGTCAAATGTG |
| MA-63 | TAGTGTCGTATTGTGTGCAGCTTGTTCTATTACCCCTAAA |
| MA-64 | AAGATGACAAATTGCTGGAGTGTCGTGGCGAGGATGTCGG |
| MA-65 | GAGCGCAGACGTCACACATTCACGGTGTTTTAGGAGAAGG |
| MA-66 | ATTAACTGTTAACTCGGTGGCAATGTTTTGTTGTTTTGCG |
| MA-67 | TTTTTTTGGTGTTACGTAGCTCGGAAAGGCAGTGGCGGTG |
| MA-68 | CAGTGTCAGAGTGTGAAGTTTAGCCTGCCCCAAAAATGCG |
| MA-69 | GAGTAGTGTGTCAAAATATTGAGTCTTTACCCGTTTCCTT |
| MA-70 | CTATGCTGTGACCTTTCATATTAAACCTGCCAACACCTGC |
| MA-71 | GCATAAACGTCGAATCTGCTTGTGACAGGCCCTACTAAAA |
| MA-72 | ATCCAAAGCGTTACTCCAAATCCGAAAATACCCAACCCGC |
| MA-73 | AGGTGTCGGACTTACTATGTGCAGCCAAATGATGATGTCT |
| MA-74 | GGTGATTTTTATCTTCCTGTTTTTCCGTGCGTTATTGGTC |
| MA-75 | ACACTCAACCAACCGGATCGCTGCACAAAACCAACTGTAA |
| MA-76 | GGGTGGTACGATGTCTTTGTTTAGAGTAGTAATCAGGTGT |
| MA-77 | GCAGGGAGTGATGTGGATGGAGACTGAGTAGGGTTTTTGC |
| MA-78 | TACTCGTCTAGCGCATTCAAATAGGGAGCACCTGGGCTGG |
| MA-79 | AGTTTAGATGAGTGGATATGTCAGTCGGGTCGTGCACATT |
| MA-80 | TGCTGCGATACTGTGGCGTGTCTCCTTGTTTAATAATACT |
| MA-81 | AGGTATCAGGAATCCAGATGGGAGGCAAAGGTCACACAAA |
| MA-82 | AGCTGTCTGTAGGGCGGAGTGCTAGGAATCAACGTTTTGC |
| MA-83 | AGGTATCAGAAAGGTATATCTCTTTTCACGGTAGCTAACT |
| MA-84 | CGCCCACGGCAGACAGGTTGTACGATATGTCAGAACAGAG |
| MA-85 | GCCATACAGATGTCAAATCCACTGCGGAGGTATAGAACGA |
| MA-86 | CAGGTCTTTTACGGACTAAAGAGGTCTTCTGTACAATATG |
| MA-87 | AGGAACACACCCCTCATTGTACAGGTGGAAAAGCGCAAAA |
| MA-88 | AGGCTTGTGTGAGTTTATTGGTTGAGGCTGCTGGTCGATG |
| MA-89 | TCCTGCATAGCGCAATACGCCCCCAACAAGAGCCAGTAG |
| MA-90 | CCGCCCCCTGCCACTATACACTGACTGCTATGGATGAACG |
| MA-91 | CACCGGCAGGCCAAATAACAGGCATCACACACACTGCAGG |
